# Hydrological interactions between oases and water vapor transportation in the Tarim Basin, northwestern China

**DOI:** 10.1038/s41598-018-31440-3

**Published:** 2018-09-07

**Authors:** Xiangyang Zhou, Wenjuan Lei

**Affiliations:** 1 College of resources and environmental engineering, Guizhou University, Guiyang, China; 2 State Key Laboratory of Hydraulics and Mountain River Engineering, Sichuan University, Chengdu, China; 3 College of Architecture & Environment, Sichuan University, Chengdu, China

## Abstract

The spatial evolution of the oasis cold-wet effects in Tarim Basin, northwestern China, are explored quantitatively by using the China Meteorological Forcing Dataset, with spatial and temporal resolution of 0.1 degree and 3 hours, respectively, and the hydrological interactions between the oases and water vapor transfer are discussed to uncover the influence of oases on regional precipitation. The results reveal that the annual precipitation exhibits an increasing trend from desert toward oasis at all four oases. However, the cold effect is not the dominating factor increasing precipitation, since the maximum increasing rate of precipitation is accompanied by the minimum decreasing rate of temperature. Indeed, water vapor transportation is more important than the cold effect. The maximum promotion of precipitation is observed in western basin, where the water vapor transfer follows the gradients of decreasing temperature and increasing humidity. Conversely, the minimum promotion in southwestern basin results from water vapor transportation following the temperature increasing. Therefore, the transfer of water vapor and its interactions with local surface conditions determine the precipitation in oasis areas. Understanding these processes is crucial to exploring the formation and spatial layout of oases, which is helpful for preventing desertification and protecting the fragile oasis ecosystem.

## Introduction

An oasis, the vegetated area of a desert, is a unique intrazonal landscape in arid and semiarid regions^1^ and is also an area where the population concentrates^2^. The Tarim Basin, covering an area of approximately 560, 000 km^2^, contains the largest desert of China in its center and many oases at its border areas^2–4^. Currently, destructive effects on oasis environments are increasingly resulting from the irrational reclamation of land and overuse of natural resources^1^. Even worse, the expansion of artificial oases has led to the degradation of natural oases and the oasis-desert ecotone, which may threaten the security and sustainable development of oases^5^. In fact, oases are comprehensively affected by climatic conditions and local hydrological effects. Therefore, exploring the hydrological interactions between oases and water vapor transportation is crucial to desertification control, oasis expansion and the security of oasis ecosystems.

Climatically, previous studies have revealed that the spatial distribution of oases can be attributed to the regional physiographic conditions and the large-scale hydrological cycle. Regarding the former, the Tarim Basin is surrounded by the mountains1, and the layout of its oases is affected mainly by the rain shadow effect of the Tibetan Plateau^6–8^. Regarding the large-scale hydrological cycle, the current understanding suggests that the reduction in westerly moisture transported from the Neotethys Ocean is highly related to the retreat of oases^9–11^.

**Figure 1:**
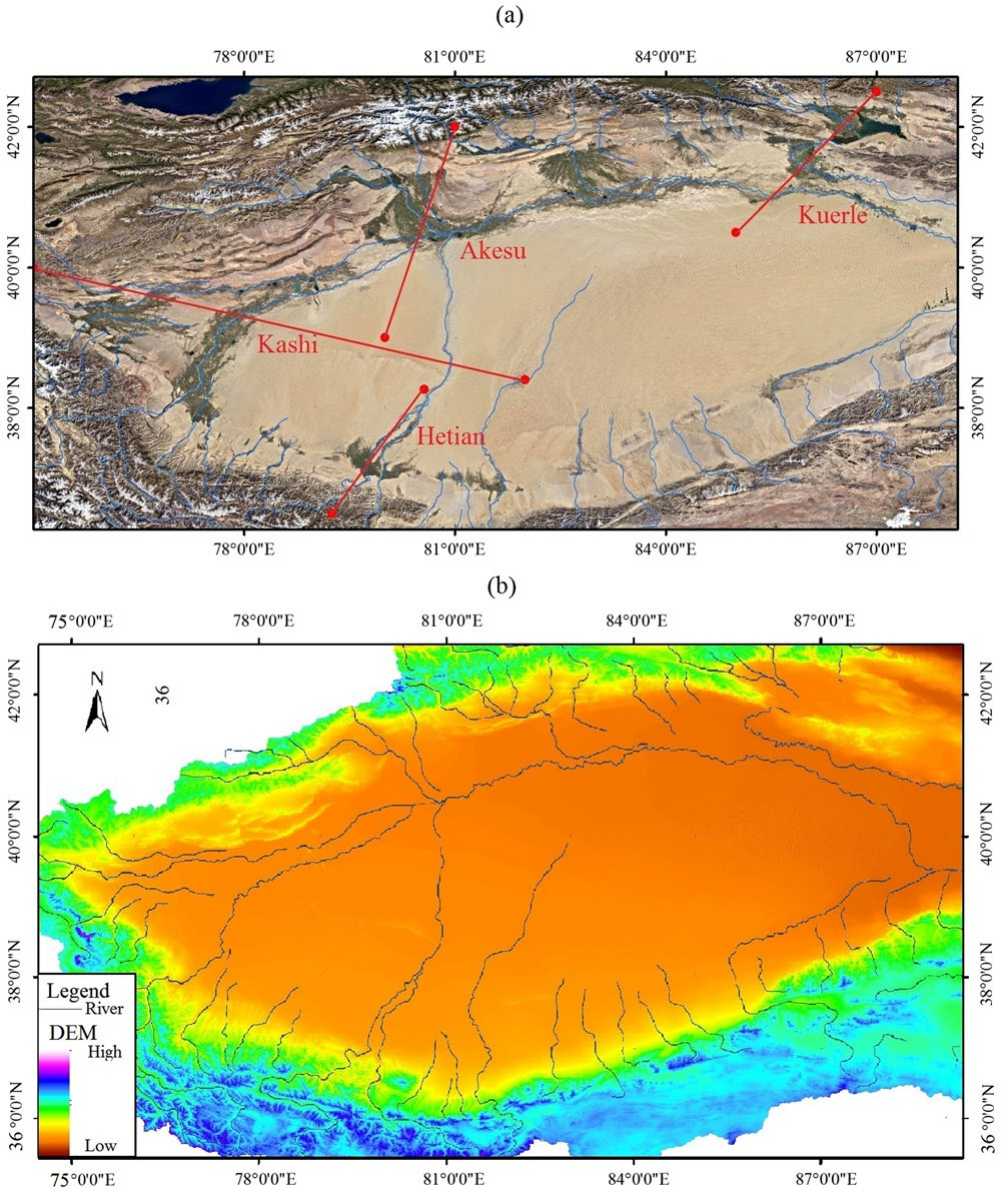
Spatial distribution of oases in the Tarim Basin and its DEM, as shown in subfigue (**a**,**b**) respectively. The remote sense image is provided by Google earth (Mapdata: Google, Landsat/Copernicus), DEM and rivers are based on Void-filled seamless SRTM data V1 (2004).

Regionally, oases are controlled by the amount of water resources. Generally, the area of an oasis is highly correlated with the amount of runoff from the mountainous areas in both the Tarim Basin and in other nearby areas^12–16^. With the development and utilization of water resources in the Tarim Basin, especially the enlargement of agriculture, the oases begin to diminish in the downstream areas and then disappear because of the shortage of water, which finally results in a spatial pattern in which oases are mainly in the upstream areas^4,17,18^, as shown in Fig. 1(a). It has been revealed that the water consumption in the irrigated oasis area is approximately 700 mm/yr, which is much higher than the local rainfall of 50–120 mm/yr^19^. This imbalance in water consumption has also been demonstrated by the relationship between climate change and the evolution of the oases^1,20^. With the continuous drought from the beginning of the Pliocene^21^, the oases have been increasingly, gradually more distributed near rivers and lakes^4^.

Apart from the above processes, the self-support mechanisms of oases are important to the size and layout of oases. First, oases serve to improve the natural conditions of a region by affecting the regional hydrometeorological factors, especially the cold-wet effect and the effect of hydrometeorological factors on increasing regional precipitation^22^ and reducing local evapotranspiration^2^. In the Tarim Basin, studies have shown that all the oases exhibit cold island effects^23^, with the highest intensity in summer, followed by autumn and spring^24,25^. A similar pattern has been observed in southern Israel, i.e., all types of vegetation create a cooling effect, and this effect is more pronounced with subtropical garden vegetation (up to 4 °C) than with local desert trees^26^. The reasons for this effect are mainly the increased absorption of latent heat by the high evapotranspiration from the oasis surface^2,27^ and the higher surface albedo of the vegetation than the desert surface^2^. In addition, increases in local water vapor sources from local evapotranspiration increase precipitation. This effect can account for 20–50% of the total ambient water vapor in humid regions^28–30^, with values up to 80% when plant transpiration is active in warm conditions^31^. Moreover, such inflow can account for up to 20% of the precipitation in arid and semiarid regions^32,33^. However, the regional hydrological effects of oases have not been revealed completely, although the cold-wet effect has been observed from sparely distributed stations. More importantly, there are few studies focusing on how the water vapor transportation driven by the large-scale hydrological cycle interacts with the local cold-wet effect of oases.

Therefore, the objectives of this study were to explore the spatial distribution and evolution of hydrological factors in the oasis-desert system on the basis of the China Meteorological Forcing Dataset with a spatial resolution of 0.1 degree and a temporal resolution of 3 hours from 1979 to 2010 and then to discuss the hydrological interactions between the cold-effect of oases and the water vapor transportation in different locations of the Tarim Basin, from which the different promotion effects of oases on local precipitation can be revealed. These processes are crucial to understanding the formation and spatial layout of oases, which can provide some useful guidelines for preventing desertification and protecting the fragile oasis ecosystems of the Tarim Basin in the long term.

## Study Area and Data

### Study area

The Tarim Basin, covering an area of approximately 560, 000 km^2^, is located in the south of Xinjiang Province in northwestern China^34^, as shown in Fig. 1. The Taklamakan Desert, which formed at least 5.3 Ma years ago, is in the center of the Tarim Basin^35^. The Taklamakan Desert, with an area of approximately 337, 600 km^2^, is the largest desert in China and the second largest shifting desert in the world^34^. The total area of the oasis is approximately 103, 900 km^27^. The main land types are farmland and shrub, with the area of 36580 km^2^ and 26940 km^2^ respectively^36^. In the desert region, the annual temperature is approximately 10 to 15 °C, with a minimum monthly temperature of −5 to −10 °C in January and a maximum temperature of 24 to 27 °C in July, according to data observed from 1960 to 2011. The annual precipitation is from 15 to 60 mm in the desert region, increasing from the east to the west. However, the annual potential evaporation is over 3200 mm^5^. With its extreme climate conditions, most of the basin is a generally unsuitable or extremely unsuitable area for human settlement, while the areas suitable or generally suitable for habitation account for approximately 10% of the total area^37^. The water vapor transportation is mainly from two sources in the summer, one from the west and the other from the southwest; in the winter, northeastern water vapor, which is mainly driven by Siberia-Mongolia high pressure, prevails^38,39^.

### Data

In this study, the quantitative results are based on the China Meteorological Forcing Dataset, which was developed by the Data Assimilation and Modeling Center for Tibetan Multispheres, Institute of Tibetan Plateau Research, Chinese Academy of Sciences^40,41^. The dataset was produced by merging a variety of data sources, including (1) CMA (China Meteorological Administration) station data, (2) Tropical Rainfall Measuring Mission, TRMM, satellite precipitation analysis data (3B42), Global Land Data Assimilation System, GLDAS, precipitation data, (3) GEWEX Surface Radiation Budget, GEWEX-SRB, downward shortwave radiation data, GLDAS downward shortwave radiation data, (4) Princeton forcing data and (5) GLDAS data. The length of the dataset is 22 years (1979–2010), and the spatial and temporal resolution was 0.1 degrees and 3 hours, respectively. The data was obtained from the website http://westdc.westgis.ac.cn/data/7a35329c-c53f-4267-aa07-e0037d913a21. More details on the dataset are given in User’s Guide for China Meteorological Forcing Dataset (http://dam.itpcas.ac.cn/data/User_Guide_for_China_Meteorological_Forcing_Dataset.htm). The accuracy of this dataset is the determined by its original data, as given in the manuscript. These original datasets are widely used to explore the hydrocoral cycles and the results, generally, are reasonable. This data set has been used to explore regional hydrological processes and the data displayed a good spatial continuity, such as the impact of lake effects on the temporal and spatial distribution of precipitation in the Nam Co basin of the Tibetan Plateau^42^, evaporation from the lake^43^, and the impact of climatic factors on permafrost in the Tibetan Plateau^44^.

## Results

### Precipitation

#### Spatial distribution of precipitation

**Figure 2:**
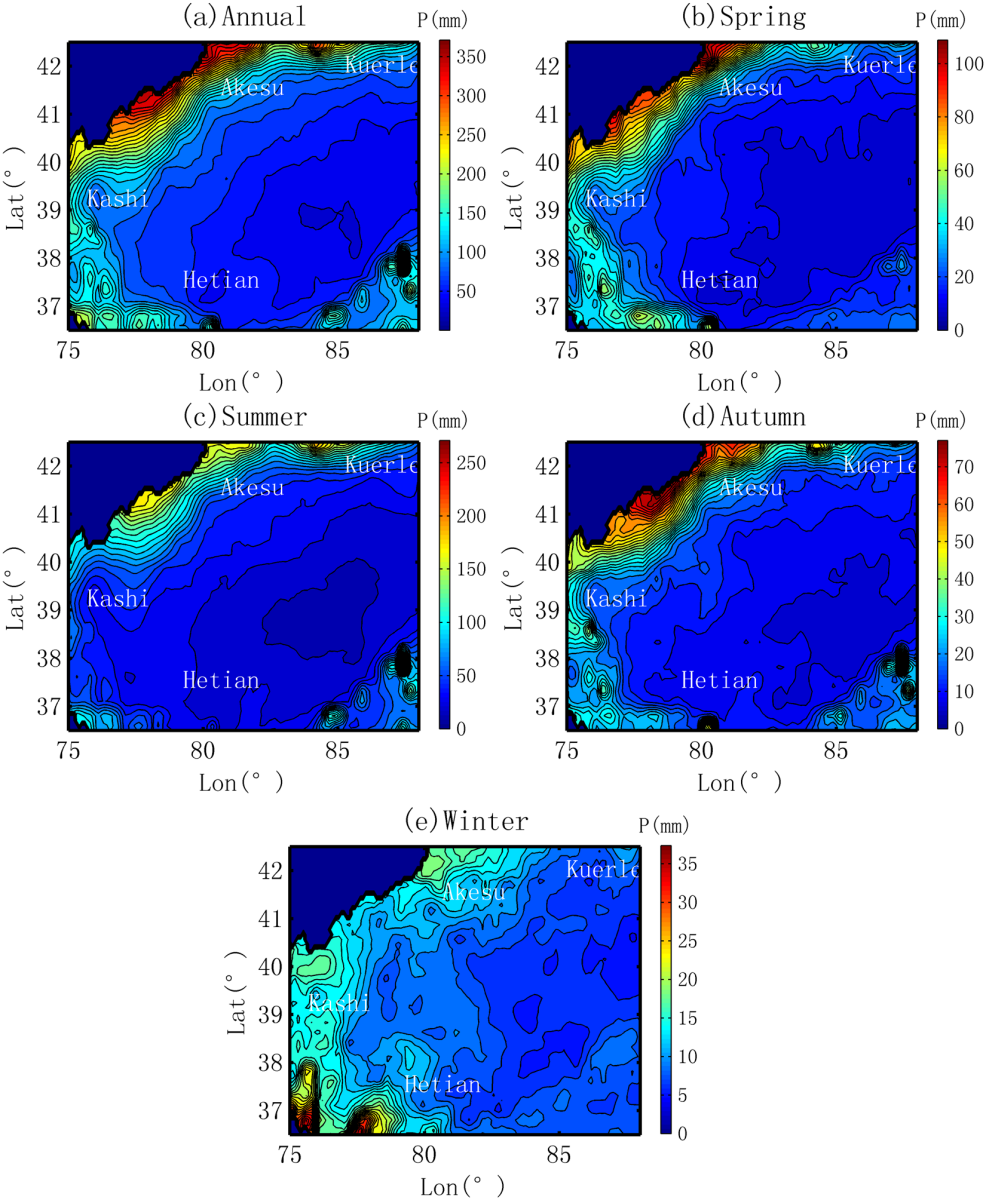
Spatial distribution of precipitation in the Tarim Basin.

The spatial distributions of precipitation in the desert-oasis system of the Tarim Basin are shown in Fig. 2. Annually, the average precipitation displays two stages, a slow increasing trend from the desert to the inner oasis and a significant uprising after the oasis-mountain boundary. The precipitation ranges from less than 50 mm to approximately 350 mm. Low precipitation is observed in the desert region, especially in the southeastern areas, with a value of 50–60 mm/yr or less. In the oasis zone, the precipitation is approximately 50–125 mm/yr.

#### Comparisons of the evolution of precipitation in different oases

**Figure 3:**
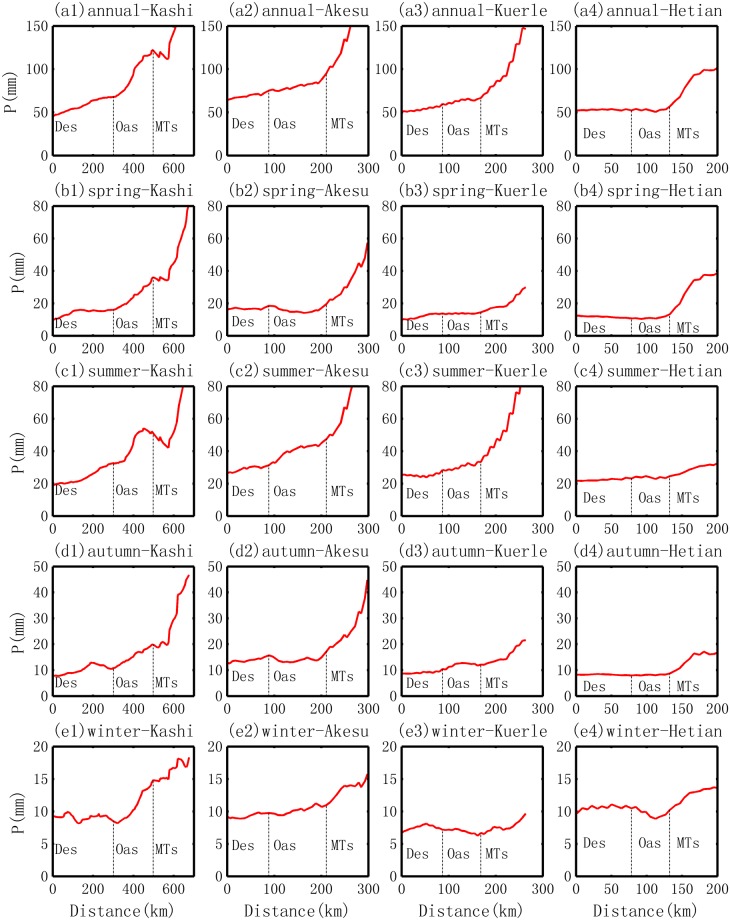
Change in precipitation from desert to oasis in different locations of the basin and in different seasons.

Following the routes in Fig. 1(a), the changes in precipitation from the desert to the oases are obtained by abstracting the corresponding values in Fig. 2. Annually, an increasing trend in precipitation is observed in all four oases, but the increment and slope vary substantially among them, as shown in Fig. 3. The maximum increase is observed in the Kashi Oasis, which exhibits a relative increment of approximately 80% (from 68 mm at the desert boundary to 121.9 mm at the mountain boundary, with an absolute increment of 53.9 mm) and an average slope of 3.2 mm/10 km. The second highest increment and rate of increase (or the slope) is observed in the Akesu Oasis. The former is an increase of 20.7 mm, accounting for about a quarter of the value of the Kashi Oasis. The latter is a rate of approximately 1.3 mm/10 km, which is much smaller than that of the Kashi Oasis. The third largest change in the oasis precipitation is observed in the Kuerle Oasis, with an average slope of 0.8 mm/10 km and an increment of 6.8 mm (or 11.4%). The smallest slope of precipitation evolution is observed in the Hetian Oasis, with a value of approximately 0.3 mm/10 km. The statistics for the general trends are summarized in Table 1.

**Table 1:** Stats for the transition of *precipitation* from desert to oasis in the Tarim Basin.

Oasis	Scale	Mean (mm)	Slope (mm/10 km)	Min (mm)	Max (mm)	Desert BDR (mm)	Mountain BDR (mm)	Average Increment (mm)	Relative increment (%)
Kashi	Annual	94.5	3.2	67.9	122.1	68.0	121.9	53.9	79.3
	Spring	24.5	1.0	16.1	36.1	16.2	35.7	19.5	120.3
	Summer	43.7	1.3	32.2	54.1	32.5	51.6	19.2	59.1
	Autumn	15.4	0.5	10.9	19.9	10.9	19.9	9.0	81.8
	Winter	10.9	0.4	8.2	14.8	8.4	14.7	6.3	74.7
Akesu	Annual	80.7	1.3	74.6	95.2	75.8	91.6	15.7	20.7
	Spring	16.1	−0.1	14.1	19.6	18.4	18.4	0.0	−0.1
	Summer	40.2	1.2	31.3	47.4	32.3	46.3	14.0	43.4
	Autumn	14.2	0.1	13.0	17.2	15.5	16.1	0.6	4.0
	Winter	10.2	0.1	9.4	11.2	9.7	10.8	1.1	11.4
Kuerle	Annual	63.1	0.8	58.7	66.6	59.2	66.0	6.8	11.4
	Spring	13.8	0.1	13.3	14.5	13.6	14.2	0.7	5.0
	Summer	30.5	0.7	28.0	33.4	28.2	33.4	5.2	18.3
	Autumn	11.9	0.2	10.2	12.8	10.3	12.0	1.6	15.7
	Winter	6.9	−0.1	6.3	7.3	7.2	6.5	−0.7	−9.7
Hetian	Annual	53.1	0.3	50.6	56.9	53.2	55.1	1.9	3.6
	Spring	11.3	0.4	10.5	13.3	10.8	12.7	1.9	17.4
	Summer	23.9	0.0	22.9	24.7	23.7	24.0	0.3	1.2
	Autumn	8.2	0.1	8.0	8.7	8.0	8.5	0.5	6.1
	Winter	9.8	−0.2	8.9	10.7	10.6	9.9	−0.7	−6.9

Note, the Desert BDR means the *precipitation* at the boundary areas between the desert and oasis; and the Mountain BDR means the precipitation at the boundary between oasis and mountains.

Seasonally, generally, the increasing trend is observed in the spring, summer and autumn. Some patterns are shown in Fig. 3(b) to (e). The first pattern is related to the main season of increasing precipitation. The maximum increment, especially the absolute increment, appears in the summer. However, a larger relative increment has also been observed in other seasons, e.g., an increase of approximately 120% in the spring in the Kashi Oasis and an increase of approximately 15% in the autumn in the Kuerle Oasis. The second pattern is related to the different trends of precipitation evolution in the winter. Similar to the trends in the other three seasons, an increasing trend is observed in the Kashi and Akesu Oases. However, the opposite trend is found in the Kuerle and Hetian Oases. This is caused by the decreasing trend in water vapor described in section 3.2.

### Water vapor content

#### Spatial patterns of mixing ratio

**Figure 4:**
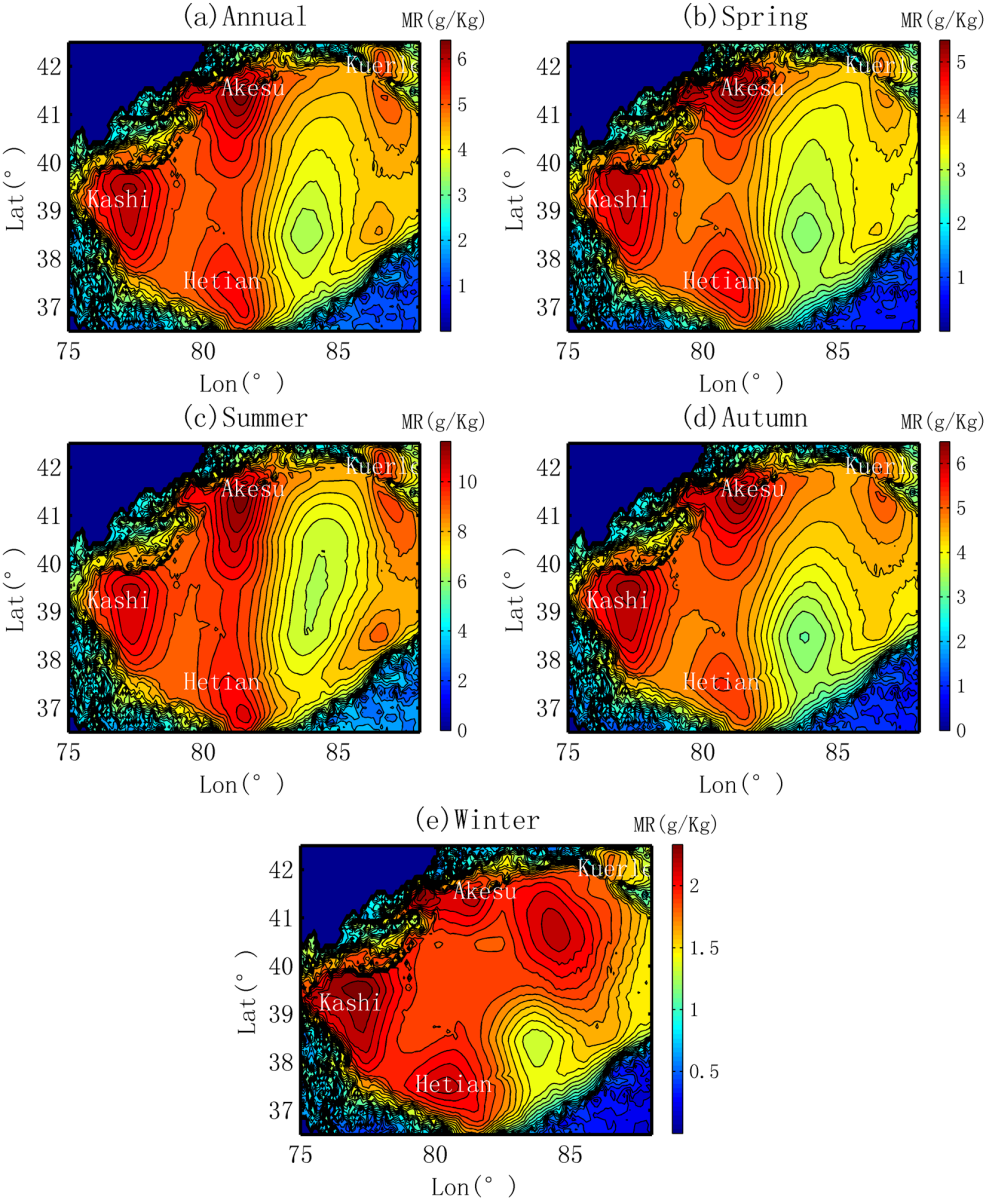
Spatial distribution of the mixing ratio (MR) in the Tarim Basin.

The spatial distribution of the water vapor content (represented by the mixing ratio, MR) generally exhibit four MR peaks in the Tarim Basin, which correspond to the Kashi Oasis in the west, the Akesu Oasis in the north, the Kuerle Oasis in the east and the Hetian Oasis in the southwest, as shown in Fig. 4. The highest MR is located in the center of the Kashi Oasis and the Akesu Oasis, followed by the MRs in the Hetian Oasis; the Kuerle Oasis shows the lowest MR peak. Quantitatively, the average annual MR ranges from 1 to 6 g/kg, and it displays three stages: intermediate values in the desert areas (3–5 g/kg), high values in the oasis areas (over 5 g/kg) and low values in the mountain areas (2–3 g/kg in the north and less than 2 g/kg in the south).

The seasonal characteristics of the MR are also compared, as shown in Fig. 4(b) to (e). Spatially, the MR does not vary substantially with the patterns at annual scale. However, there are several differences. In the summer, a high MR belt is observed along the Tarim River. This can be interpreted as a result of the more intensive evaporation from the free water surface of the Tarim River, as shown in Fig. 2. The second is that the MR peak is sometimes not located over and oasis. In the summer, the MR peak is located over the desert area of the Hetian Oasis; in the winter, compared with its annual location and locations in the three other seasons, the MR peak in the Kuerle Oasis moves toward the southwest. This phenomenon is caused by the hydrological cycle that will be discussed in section 4.

#### Comparisons with the evolution of the MR in different oases

**Figure 5:**
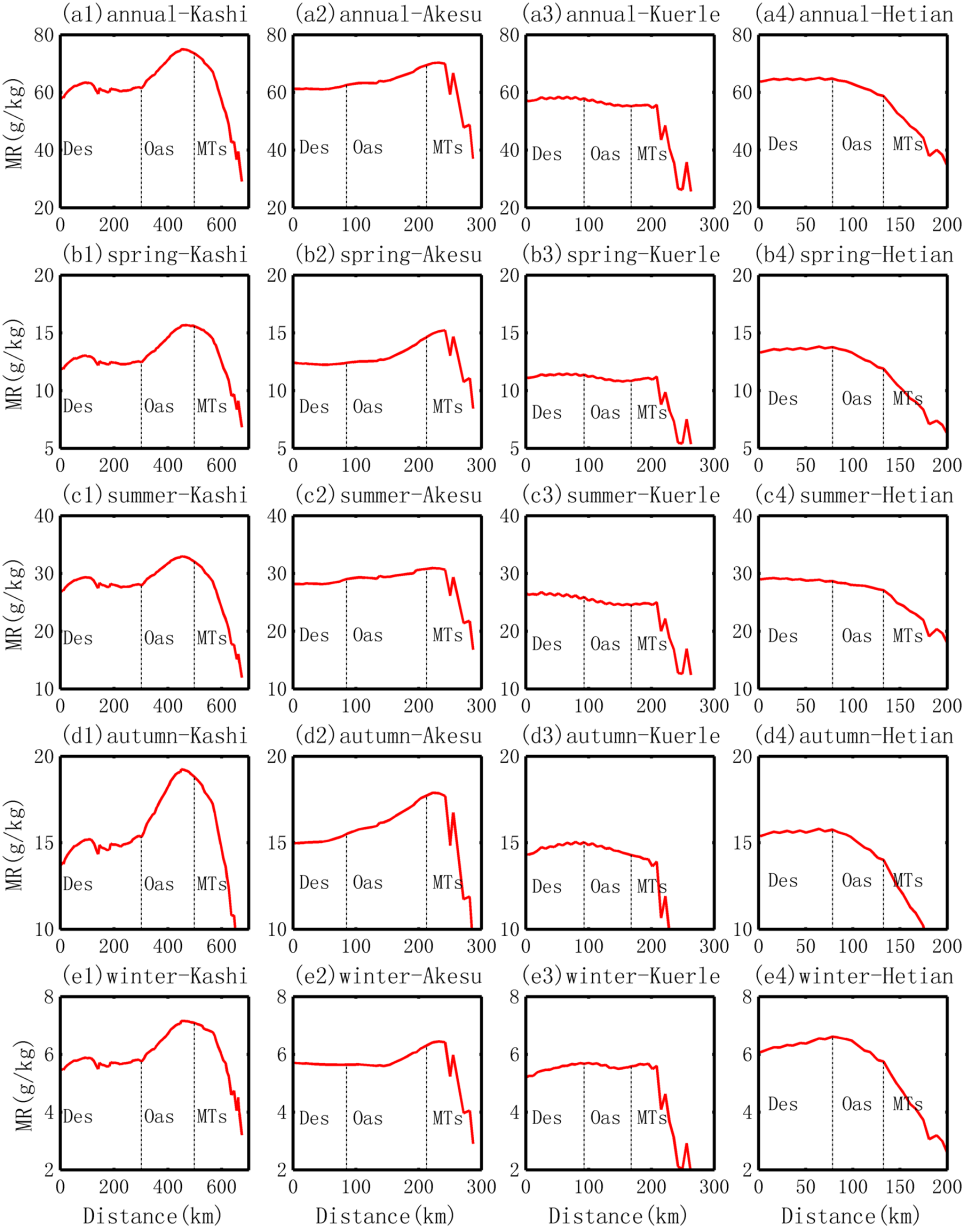
Change in water vapor content represented by the mixing ratio (MR) from the desert to inner oasis in different locations of the basin and at different seasons.

Similarly, following the routes in Fig. 1, the changes in MR from the desert border to the inner oasis are obtained and are shown in Fig. 5. The evolutions of the MR in the four oases are as follows.

1. Ranking of the relative increment in MR. The relative increment (annual scale) is approximately 20%, 10%, −4% and −10% for the oases in Kashi, Akesu, Kuerle and Hetian, respectively. This order is the same as that of the precipitation change. Seasonally, the relative increment does not vary substantially for each oasis, none of the four seasons demonstrate the opposite trend. Generally, a smaller value (increasing or decreasing rate) is observed in the summer. The statistics for the general trends in MR are given in Table 2.

**Table 2:** Stats for the transition of *water vapor content* (mixing ratio, MR) from desert to oasis in the Tarim Basin.

Oasis	Scale	Mean (g/kg)	Slope (g.kg^−1^.10 km^−1^)	Min (g/kg)	Max (g/kg)	Desert BDR (g/kg)	Mountain BDR (g/kg)	Average Increment (g/kg)	Relative increment (%)
Kashi	Annual	70.1	0.7	61.5	75	62	73.7	11.7	18.9
	Spring	14.5	0.2	12.5	15.7	12.6	15.6	3	24.2
	Summer	31.2	0.2	28	33	28.2	32.2	4	14.2
	Autumn	17.9	0.2	15.3	19.2	15.5	18.9	3.4	21.9
	Winter	6.6	0.1	5.8	7.2	5.8	7.1	1.3	22.3
Akesu	Annual	65.1	0.5	62.6	69.4	62.8	69.1	6.3	10.1
	Spring	13.1	0.2	12.4	14.6	12.4	14.5	2	16.4
	Summer	29.7	0.1	29	30.8	29.1	30.7	1.6	5.6
	Autumn	16.5	0.2	15.5	17.7	15.6	17.6	2.1	13.3
	Winter	5.8	0.1	5.6	6.3	5.6	6.3	0.6	10.8
Kuerle	Annual	56.3	−0.4	55.2	58	57.6	55.4	−2.2	−3.8
	Spring	11	−0.1	10.8	11.4	11.3	10.9	−0.4	−3.9
	Summer	25	−0.2	24.5	25.9	25.6	24.6	−1.0	−4.0
	Autumn	14.7	−0.1	14.3	15	15	14.3	−0.6	−4.2
	Winter	5.6	0	5.5	5.7	5.7	5.6	−0.1	−2.2
Hetian	Annual	62.1	−1.1	58.8	64.9	64.5	59	−5.5	−8.5
	Spring	12.9	−0.4	11.9	13.8	13.7	12	−1.7	−12.7
	Summer	27.9	−0.3	27.1	28.7	28.5	27.2	−1.3	−4.6
	Autumn	15	−0.3	14	15.8	15.7	14.1	−1.6	−10.3
	Winter	6.3	−0.2	5.8	6.6	6.6	5.8	−0.8	−12.5

Note, the Desert BDR means the water vapor content (mixing ratio) at the boundary areas between the desert and oasis; and the Mountain BDR means the *water vapor content* at the boundary between oasis and mountains.
2. Increase versus decrease. As shown in Fig. 5, two oases (Kashi and Akesu) exhibit an increasing trend in MR, while the other two oases exhibit a decreasing trend in MR. This implies that the water vapor content does not always accumulate over the oasis. Sometimes, the content is larger in the desert in the region close the boundary between the oasis and desert; this is the case for the peak in MR in the Kuerle and Hetian Oases.

### Temperature

#### Spatial distribution of temperature

**Figure 6:**
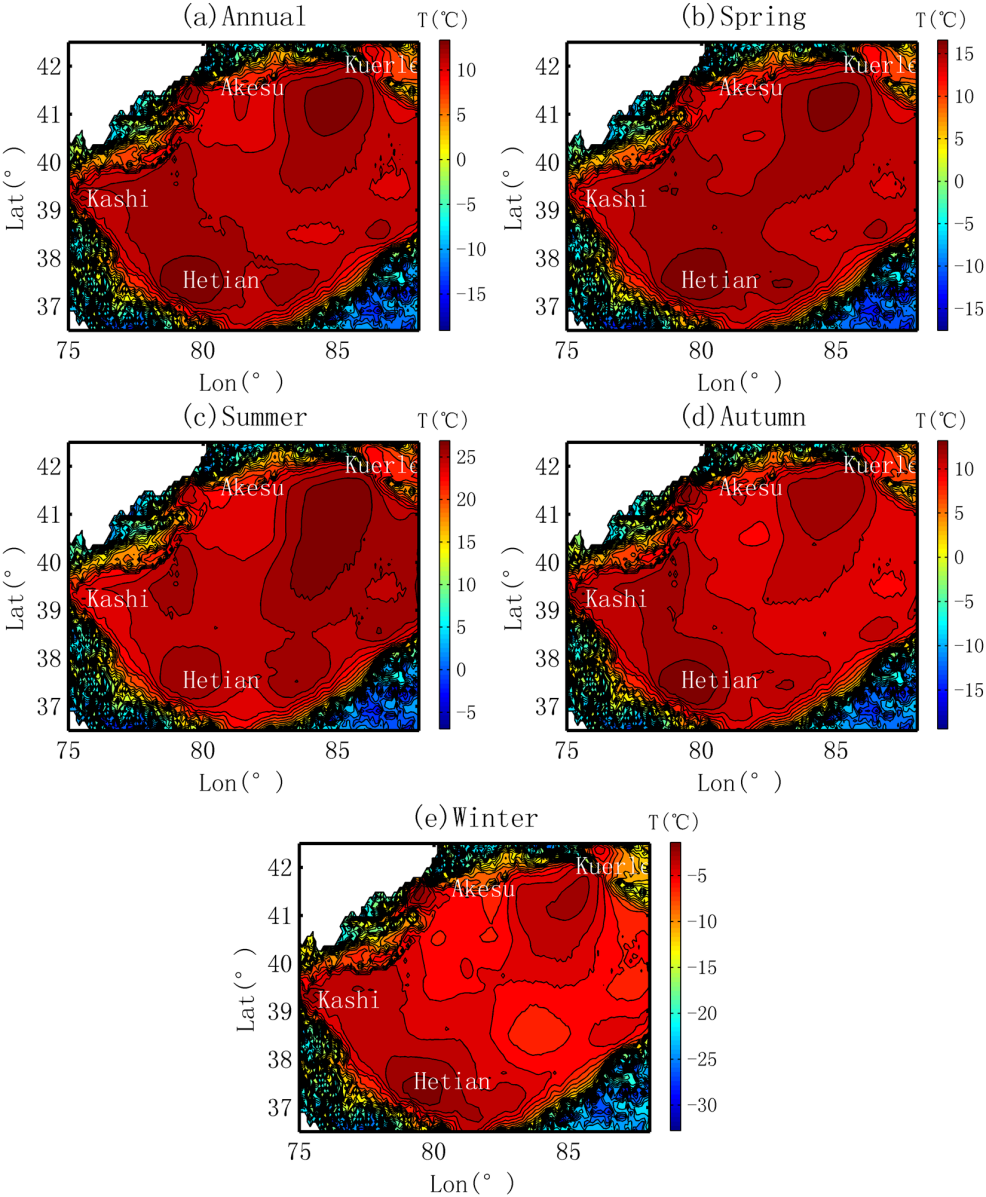
Spatial distribution of Temperature in Tarim Basin.

The spatial distributions of temperature in the Tarim Basin are shown in Fig. 6. At the annual scale, generally, the cold effect is observed. The temperature shows a mean of over 10 °C at the boundary between the oasis and desert, and then it gradually drops to 8 to 10 °C in the oasis areas. The lowest temperature is observed in the mountain areas where the terrain lifts substantially.

Seasonally, similar to the spatial patterns in MR, oases cold effects are observed in the spring, summer and autumn. In general, the temperature exhibits a decreasing trend from the desert to oasis. It should be noted that the average temperature in the summer is approximately 22–28 °C in the basin. This means a decrease in temperature of a couple of centigrade will lead to a substantial decrease in the water vapor holding capacity of the atmosphere because of the exponential relationship between them. In the winter, however, an opposite trend, or the heat effect, is observed in the Kashi and Hetian Oases. These peaks correspond well with the locations of the oases. A similar trend has also been observed by other studies, and the reason underlying the trend is mainly the consumption of fuels driven by human activities^45^. For example, fast developments in tourism and civilization and increases in population result in a much higher rate of increase of the temperature in oasis areas^46^.

#### Comparisons of the evolution of temperature in different oases

**Figure 7:**
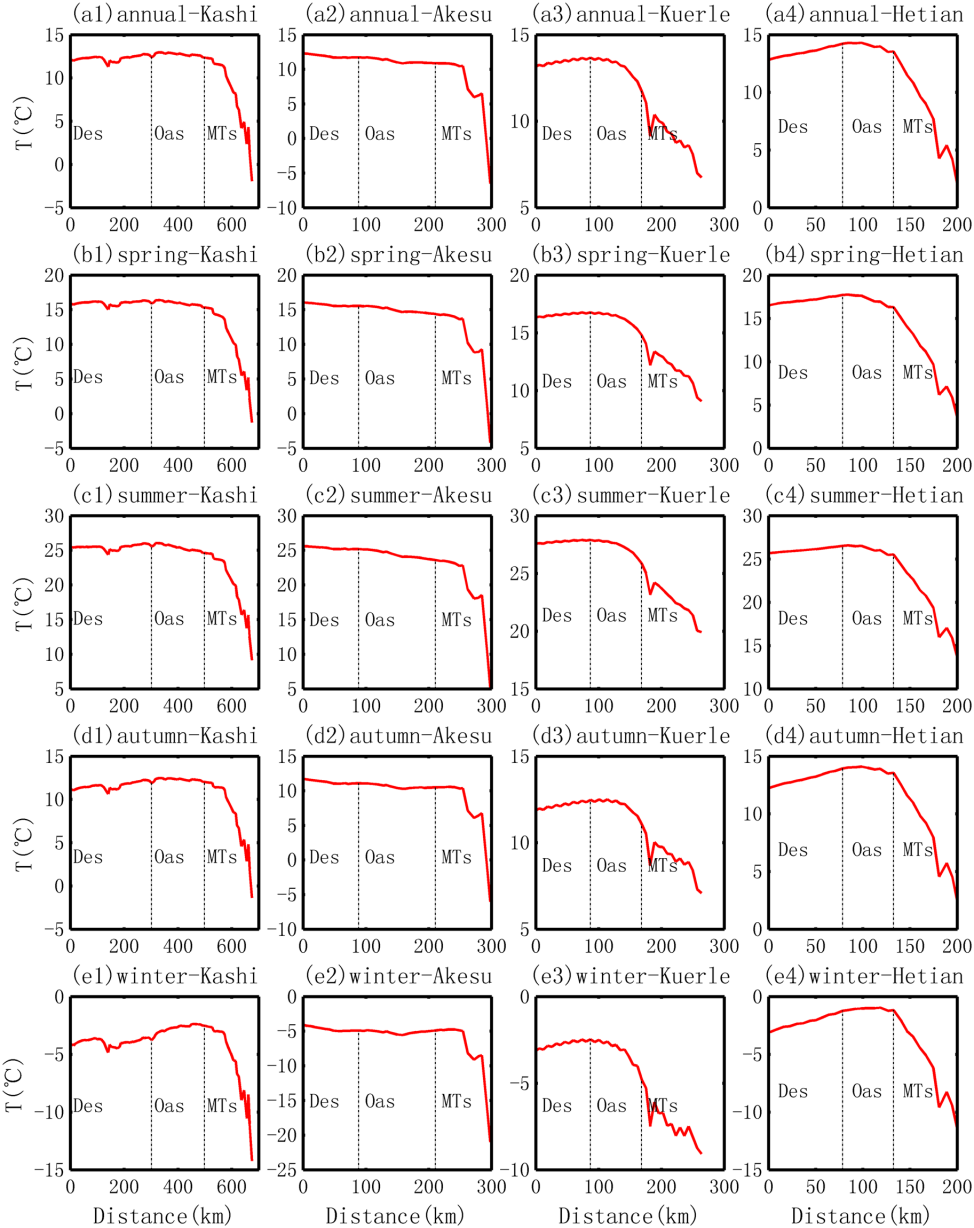
Change in temperature from the desert to the oasis in different locations of the basin and in different seasons.

Intensity of cold effect. The cold effect displays different levels in the four oases, as shown in Fig. 7. The average annual levels are −1.6, −0.8, −0.6 and −0.1 °C for the Kuerle, Akesu, Hetian and Kashi Oases, respectively. On the other hand, the decreasing rate shows different patterns, i.e., it demonstrates a maximum of −0.2 °C/10 km in the Kuerle Oasis, followed by the rate of −0.15 °C/10 km in the Hetian Oasis, the rate of −0.08 °C/10 km in the Akesu Oasis and the minimum rate of −0.02 °C/10 km in the Kashi Oasis Seasonally, more intensive cold effects are observed in the summer and spring, and the decrease in temperature can be over 2 °C. More details are summarized in Table 3. Here, it is found that the intensity of the cold effect is opposite to the increasing rate of precipitation in the four oases. This means that the greatest cold effect is not accompanied by the highest increasing rate of precipitation, such as the case of the Hetian Oasis, while the highest increasing rate of precipitation is found in the Kashi Oasis, which has the lowest cold effect. Hence, it can be inferred that the cold effect is not the main factor that increases the local precipitation of an oasis area.

**Table 3:** Stats for the transition of temperature from desert to oasis in the Tarim Basin.

Oasis	Scale	Mean (°C)	Slope (°C.10 km^−1^)	Min (°C)	Max (°C)	Max-Min (°C)	Desert BDR (°C)	Mountain BDR (°C)	Average Increment (°C)
Kashi	Annual	12.7	−0.02	12.4	13.0	0.6	12.5	12.4	−0.1
	Spring	16.0	−0.04	15.4	16.4	1.0	16.0	15.4	−0.6
	Summer	25.4	−0.07	24.6	26.1	1.5	25.7	24.6	−1.0
	Autumn	12.3	−0.01	11.9	12.5	0.6	12.1	12.0	0.0
	Winter	−2.7	0.05	−3.8	−2.4	1.4	−3.6	−2.5	1.1
Akesu	Annual	11.2	−0.08	10.9	11.7	0.8	11.7	10.9	−0.8
	Spring	15.0	−0.11	14.4	15.6	1.2	15.5	14.4	−1.1
	Summer	24.4	−0.14	23.6	25.2	1.6	25.2	23.7	−1.5
	Autumn	10.7	−0.06	10.3	11.1	0.8	11.1	10.5	−0.6
	Winter	−5.1	−0.01	−5.5	−4.9	0.7	−4.9	−4.9	0.0
Kuerle	Annual	13.1	−0.20	11.7	13.7	2.0	13.6	12.0	−1.6
	Spring	16.2	−0.21	14.8	16.8	2.0	16.7	15.1	−1.7
	Summer	27.3	−0.22	25.9	27.9	2.0	27.9	26.1	−1.8
	Autumn	12.1	−0.14	11.1	12.5	1.4	12.4	11.3	−1.1
	Winter	−3.1	−0.24	−4.8	−2.5	2.3	−2.5	−4.4	−1.9
Hetian	Annual	14.0	−0.15	13.5	14.3	0.8	14.3	13.5	−0.7
	Spring	17.2	−0.29	16.3	17.8	1.5	17.7	16.3	−1.4
	Summer	26.2	−0.21	25.5	26.6	1.1	26.6	25.5	−1.1
	Autumn	13.9	−0.09	13.5	14.1	0.6	14.0	13.5	−0.5
	Winter	−1.1	0.01	−1.2	−0.9	0.3	−1.2	−1.0	0.20

Note, the Desert BDR means the *temperature* at the boundary areas between the desert and oasis; and the Mountain BDR means the *temperature* at the boundary between oasis and mountains.

(2) Heat effect in the winter. Two oases exhibit a heat effect. The more intensive heat effect is observed in the Kashi Oasis, with the slope of 0.05 °C/10 km and an increment of 1.1 °C, while a weaker effect was observed in the Hetian Oasis, with a slope of 0.01 °C/10 km and an increment of 0.2 °C. In the Akesu Oasis, the temperature first decreases and then increases, which makes the slope almost zero in the winter. However, in the Kuerle Oasis, it displays a more intensive cold effect, with a slope of −0.24 °C/10 km and an increment of −2 °C. In the Kuerle Oasis, the substantial decrease in temperature can be interpreted as the result of two aspects. The first is the weaker local human activities. According to the local government statistics, the population size of 30 thousand in the Kuerle Oasis is much smaller than that of the other three oases. The second is the peak of water vapor is not observed over the oasis area. As shown in Fig. 4(e), the water vapor shows a substantial decreasing pattern from the desert to the inner oasis. Water vapor is the greenhouse gas that would make the temperature higher. The main reasons for the spatial distribution of water vapor are correlated with the hydrological cycle and water vapor sources that are discussed in section 4.2.

## Discussion

The results above reveal that the oasis promotes greater local precipitation, but this effect varies substantially in different locations of the oasis. The larger cold effect accompanied by a smaller increasing rate of precipitation means that the main influencing factor promoting precipitation is not the local oasis cold effect. The good correlation between the water vapor content and precipitation reflects the oasis wet effect plays an important role in regional precipitation, but the mechanism of this role is not clear, especially how the local water vapor interacts with the large-scale cycle. These questions are discussed in the following sections.

### Influence of terrain on local precipitation

**Figure 8:**
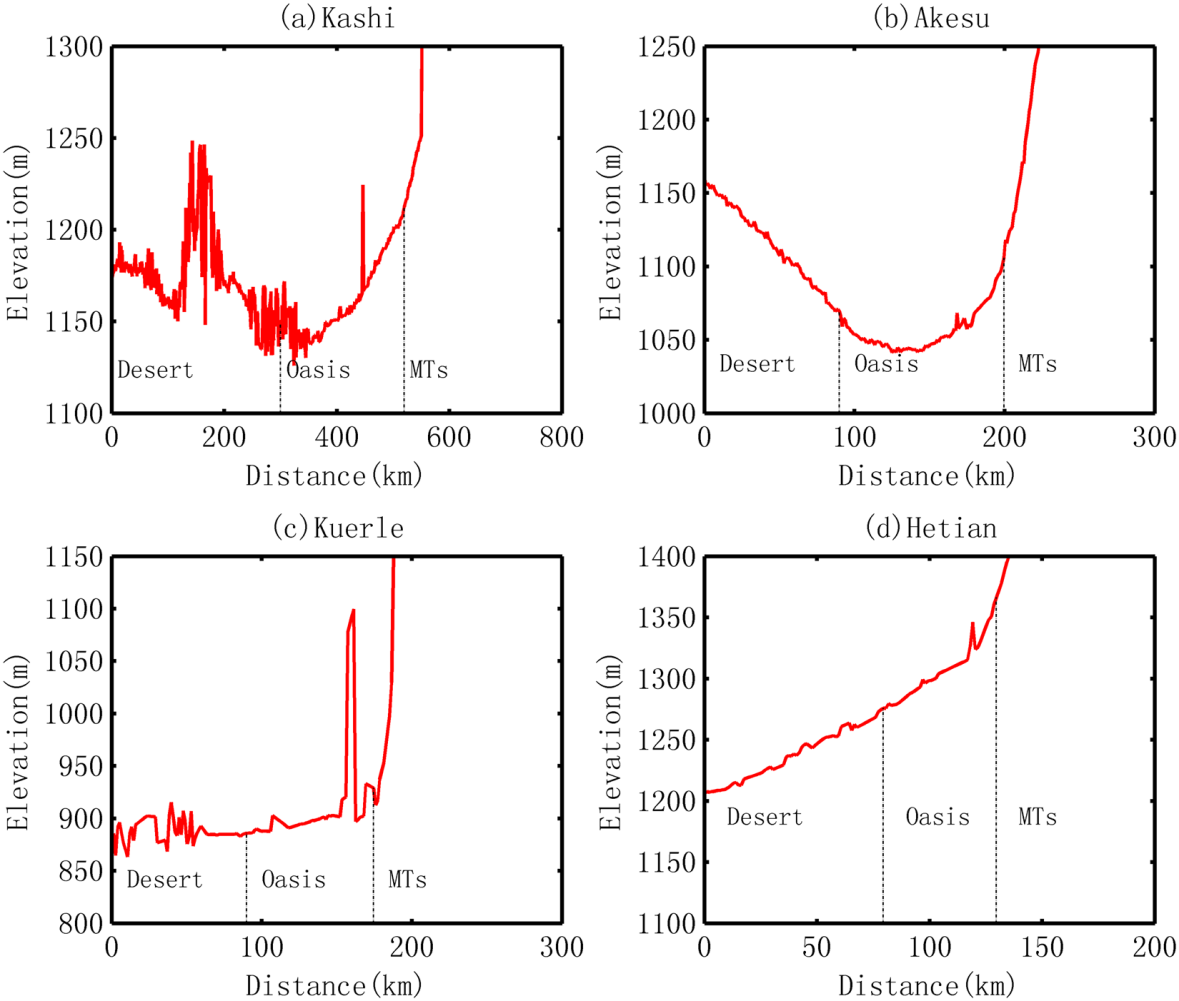
Elevation profiles for typical oases in the Tarim Basin.

Apart from the vegetation cover, the significant uplift of the terrain would lead to a sharp decrease in temperature and result in a substantial increase in precipitation at the local scale. On the basis of DEM, the elevations of the selected routes from the desert to oasis are obtained. Generally, the elevation displays an increasing trend in the four oases, but the increase is not substantial and the increments are less than 100 m for all the selected profiles, as shown in Fig. 8. In the Kashi Oasis, the elevation increment is approximately 70 m (from 1150 m 1220 m); in the Akesu Oasis, the minimum elevation is approximately 1050 m, and the highest is approximately 1130 m, with an increment of 80 m; in the Kuerle Oasis, the elevation ranges from 880 m to 930 m, with the exception of a small hill that increases the elevation to approximately 1100 m; in the Hetian Oasis (Fig. 8(d)), the increment of elevation is approximately 95 m, from 1275 to 1370 m. Here, the increments in altitude are similar among the four selected oasis profiles, which show that the total influence of terrain on local temperature is almost the same across the oases. In fact, the widths of the Kashi, Akesu, Kuerle and Hetian Oases are approximately 210, 120, 80 and 55 km, respectively, as shown in Fig. 8. This indicates that the increase rate is ordered from Hetian>Kuerle>Akesu>Kashi. More specifically, according to the relationship between temperature and elevation, an increase in height of 80–100 m would lead to a decrease in temperature by up to 0.5–0.6 °C. The data about temperature changes given in Table 3 shows that the decrease in temperature is much larger than the influence of the local DEM. In the summer, for example, the temperature decreases by approximately 1.4, 1.6, 2.0 and 1.4 °C in the oasis areas of Kashi, Akesu, Kuerle and Hetian, respectively. Hence, the possibility that most of the promotion of precipitation resulted from the local terrain can be safely excluded.

### Hydrological cycle and water vapor sources

**Figure 9:**
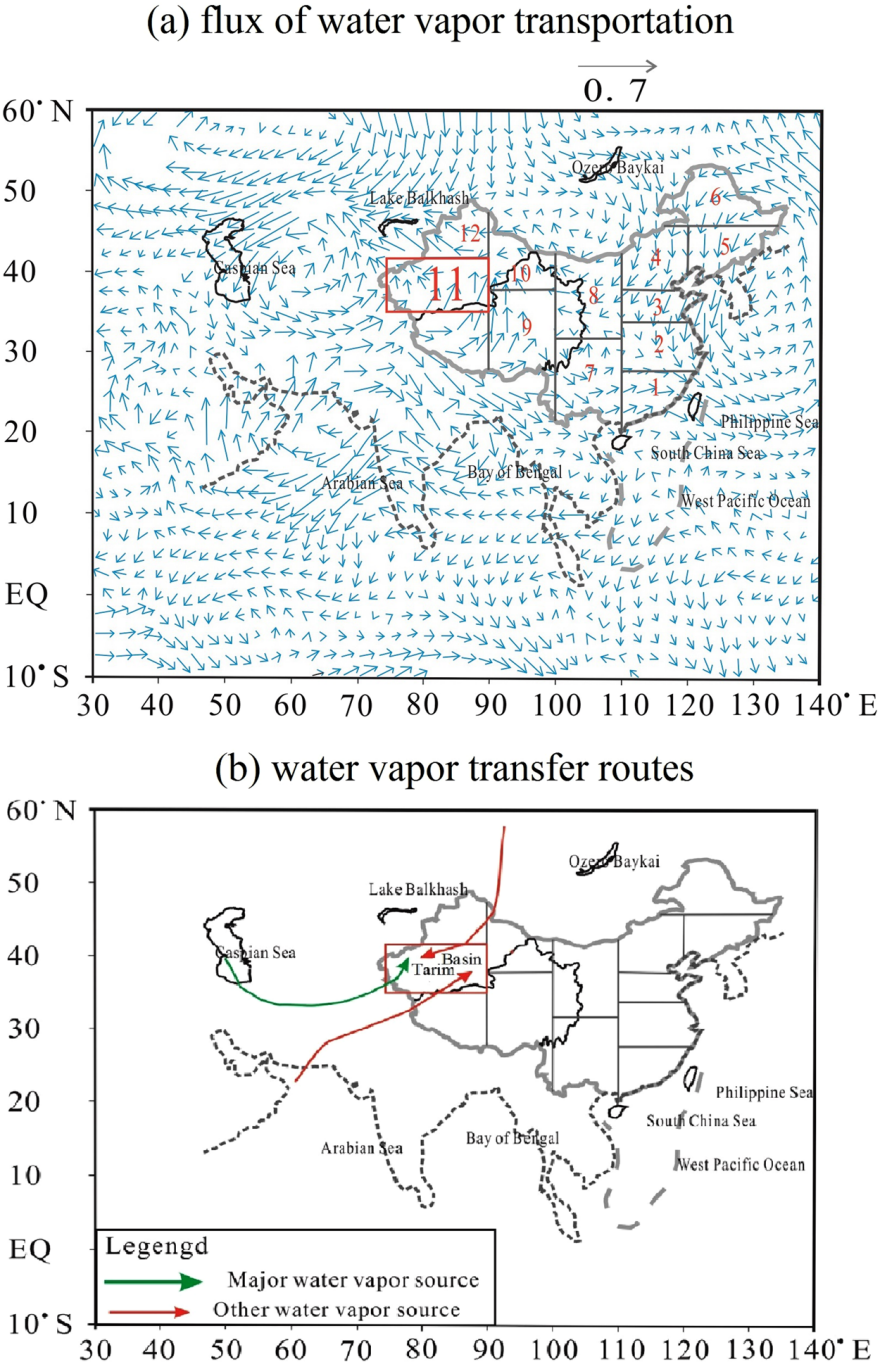
Water vapor sources in the summer and their transfer routes in the Tarim Basin based on the results calculated by Shen^48^ using the NCEP/NCAR reanalysis dataset during 1951–2006.

Previous studies have calculated the water vapor fluxes for each month on the basis of NECP reanalysis data^47^, and they have also revealed that the flux in the summer generally dominates the precipitation in the Tarim Basin^47,48^. Here, the fluxes of water vapor and their transfer routes in the summer are shown in Fig. 9. Similar water vapor fluxes and transfer routes have also been reported by other studies^49,50^. In the Tarim Basin, three water vapor sources are identified. The first one (the main source) is from the northern Caspian Sea, from which water vapor enters the Tarim Basin in a southeastern direction. The second water vapor source is from the northern Arabian Sea; water vapor from this source crosses the western Tibetan Plateau and finally enters the Tarim Basin with a southwestern direction. The third source is the eastern part of Lake Balkhash; vapor from the lake enters the basin in the northeastern area with a northeastern direction. In the winter, the water vapor is dominated by continental sources primarily from Siberia in the north and the Caspian Sea and southwestern Tibetan Plateau in the west^37^. Water vapor from the Atlantic and the Arctic Oceans, which has an important influence on the precipitation of Xinjiang, has also been observed in the Tarim Basin^39^.

Based on the water vapor sources and transfer routes above, it is obvious why water vapor content displays an increasing trend from the desert to the inner oases of Kashi and Akesu, while displaying the opposite trend in the Kuerle and Hetian Oases. In the Kashi Oasis, located in the western basin, the water vapor transfer route follows the desert-oasis direction; the main source of water vapor is the northern Caspian Sea in summer^48^ and Siberia in the winter^38,39^. In the Akesu Oasis, the water vapor transfer is also in the desert-oasis direction; water vapor from the northern Arabian Sea enters the basin from the southwestern direction in summer^46^, while there is some angle to the Siberian water vapor source in the winter. Here, both situations result in the local water vapor from evapotranspiration accumulating in the oasis areas and showing a peak over the oasis. In contrast, in the Hetian Oasis, the prevailing wind in the summer is from the oasis to the desert (southwestern direction), and the local water vapor is blown to the downwind areas. In the Kuerle Oasis, the main water vapor transfer route is opposite to the desert-oasis direction in both the summer and winter, which results in the water vapor accumulating in the downwind areas and displaying a decreasing trend in water vapor from the desert to inner oasis. This can also reflect why no heat effect is observed in the winter for the Kuerle Oasis, i.e., the local water vapor accumulating in the downwind area produces a greenhouse effect, showing a decreasing trend from the desert to oasis.

### Hydrological interactions

By overlapping the water vapor transfer routes, the spatial patterns of the local climatic factors and the oasis distributions, different characteristics are observed for the four main oases.

- In the Kashi Oasis, the water vapor transportation almost follows the desert to oasis route. That is, the water vapor transfer route is the same as the decreasing trend in temperature and the increasing trend in vapor, which leads to a substantial increase in precipitation. Here, we can conclude that the large-scale hydrological cycle interacts positively with the local cold effect and water vapor source from the oasis, a pattern similar to that of the orographic rainfall mechanisms.
- In the Akesu Oasis, the water vapor transfer route displays an acute angle to the desert-oasis profile. Furthermore, the water vapor transfer route is almost parallel to the contour lines of temperature. Hence, the promoting effect on precipitation resulting from the local water vapor sources and cold effect is smaller than that of the Kashi Oasis.
- In the Kuerle Oasis, however, the water vapor transportation routes are almost opposite to the desert-oasis direction in both the summer and winter. This can explain the abnormal spatial patterns in both temperature and water vapor content. Finally, the comprehensive interactions among the local water vapor distribution, temperature field and the water vapor transportation have smaller positive effects on increasing precipitation.
- In the Hetian Oasis, a similar pattern to that of the Kuerle Oasis is observed. The water vapor transportation route is opposite to the desert-oasis direction. Ever worse, the desert-oasis direction is opposite to the direction of the two main water vapor sources in the summer. The peaks in water vapor can also be observed in the downwind areas of the oasis. Apart from that, the increase in temperature leads to a greater water vapor holding capacity. In the summer, an approximately 1.6 °C increase in temperature would increase the water vapor holding capacity by 30% based on the exponential model. Hence, the promoting effect on precipitation is the lowest in the Hetian Oasis.

**Figure 10:**
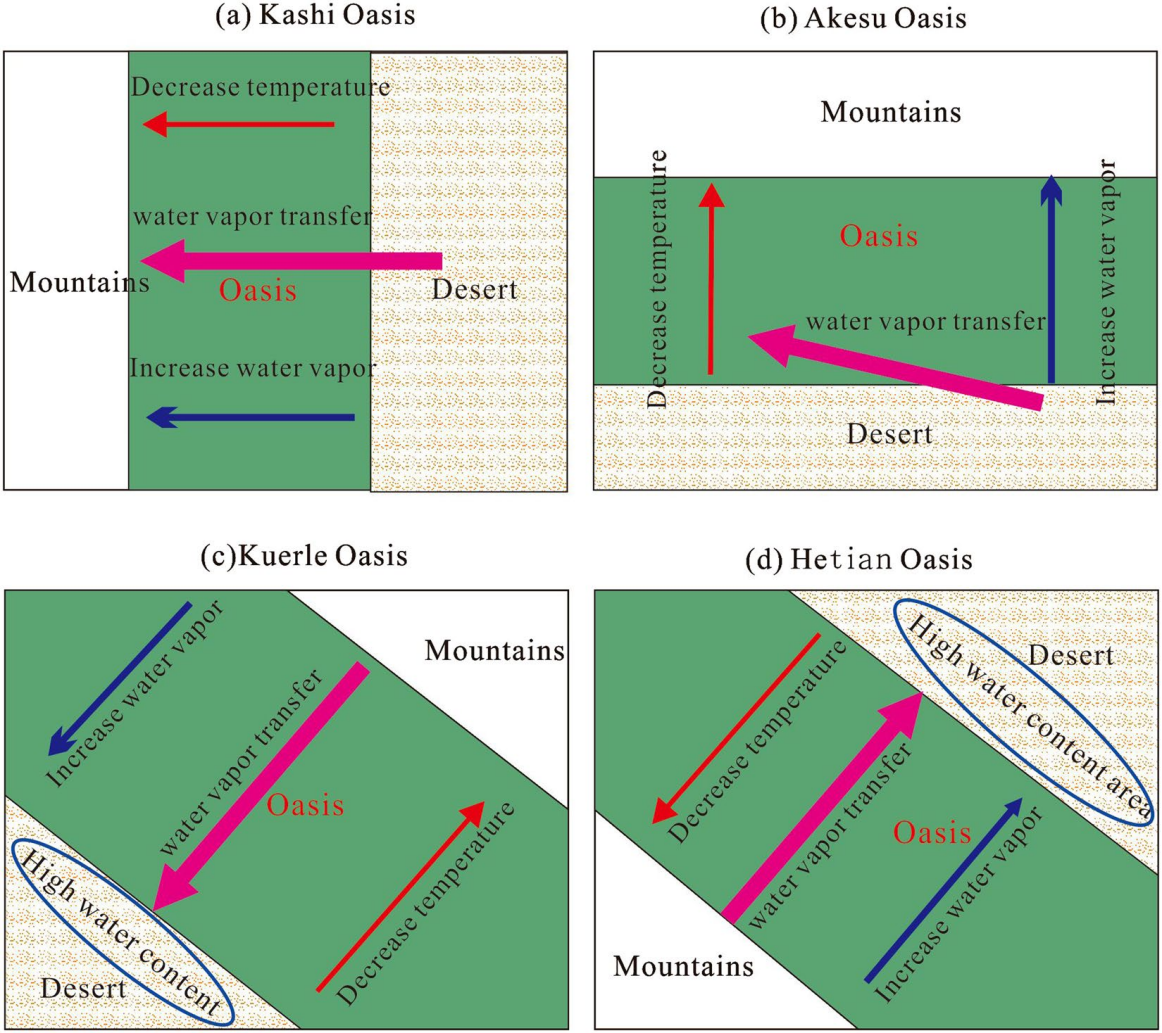
The illustration of the hydrological interactions between oasis cold-wet effect and water vapor transportation in the Tarim Basin.

Therefore, the water vapor transfers driven by the hydrological cycle and their interactions with the local environmental factors controlled by oasis are clear, as illustrated in Fig. 10. (1) When the directions of water vapor transportation, decreasing trend in temperature and increase in local water vapor are the same, the most significant promoting effect on precipitation results (with an average slope of 3.16 mm/10 km annually), as shown by the patterns of the Kashi Oasis. (2) When the route of the water vapor transportation displays an angle to the decreasing trend in temperature and increase in water vapor content, the oasis-promoted increase in precipitation decrease (with an average slope of 1.27 mm/10 km annually), as illustrated by the patterns of the Akesu Oasis. (3) When the direction of water vapor transportation is opposite to the desert-oasis direction, the water vapor does not accumulate in the oasis area, and the peaks of water vapor are observed in the downwind areas of the oasis (in the desert), as illustrated by the patterns of the Kuerle and Hetian Oases. In the Hetian Oasis, the main water vapor transfers are opposite to the desert-oasis direction in the summer and result in the lowest increasing rate in precipitation.

Significance of hydrological interactions. Contemporarily, the destructive effects on the oasis environment of the Tarim Basin are increasingly resulting from the irrational reclamation of land and overuse of natural resources^1^. The water transfer project can effectively prevent desertification. However, the expansion of artificial oases has led to the degradation of natural oases and the oasis-desert ecotone^50^. According to the different interacting mechanisms between the local oasis cold-wet effect and large-scale water vapor transfer of the four oases, it can be concluded that the western basin is the most appropriate place to prevent desertification, recover vegetation and transfer water. The cold-wet effect and its interactions with monsoons will increase the local precipitation of the western oasis much more than that of the other three oases and provide better natural conditions for plant growth. In contrast, the southern basin is the most challenging area to preventing desertification and oasis expansion. The reason for this is mainly that the cold effect of an oasis is opposite to the trend in water vapor increase when an oasis is not wide enough. The local water vapor from evapotranspiration is blown away and accumulates over the desert downwind of the oasis. The rise in temperature would increase the water vapor holding capacity by 30% based on the exponential model; thus, the local water vapor cannot effectively change to precipitation.

## Conclusions

Based on the local hydrological effects of the oases and their interactions with water vapor transportation in the Tarim Basin, the following conclusions are provided.

1. The spatial evolutions of hydrological factors exhibit different patterns from the desert toward the oases in different locations of the basin. Precipitation, annually, exhibits an increasing trend in all four oases, with rates of increase of 3.16, 1.27, 0.83 and 0.34 mm/10 km for the Kashi Oasis in the western basin, the Akesu Oasis in the northern basin, the Kuerle Oasis in the northeastern basin and the Hetian Oasis in the southwestern basin, respectively. Correspondingly, the changing rates in their water vapor contents are 0.69, 0.53, −0.35 and −1.15 g.kg^−1^. However, the changing rates in temperature show the opposite trend in the four oases, with the values of −0.07, −0.14, −0.22 and −0.21 °C °C/10 km for the Kashi, Akesu, Kuerle, and Heitian Oases, respectively. That the larger decreasing rates in temperature were accompanied by the smaller increasing rates of precipitation implies that the cold effect of the oasis is not the dominating factor that promotes the regional precipitation.
2. The oasis-promoted increase in local precipitation is mainly dominated by water vapor transportation and the interactions of water vapor with the oasis cold-wet effect. From the desert to the oases, the promotion of precipitation reaches a maximum when the water vapor transfer route follows a positive gradient of water vapor content and a negative gradient of temperature, e.g., at the Kashi Oasis. If there is an angle between the water vapor transfer route and the decreasing temperature and increasing water vapor gradients, the promotion effect decreases, e.g., at the Akesu Oasis. However, the promotion is the lowest when the transfer is opposite to the desert-oasis direction because the water vapor transportation follows an increasing temperature gradient and accumulates over desert. The exponential increase in the water vapor holding capacity of the atmosphere with rising temperature leads to less water vapor change into precipitation and promotes least local precipitation, e.g., at the Hetian Oasis, where the peak of water vapor observed in the desert at the downwind direction.

Therefore, the different interacting mechanisms between the local oasis cold-wet effect and water vapor transportation result in the promotion of local precipitation, which varies substantially in the different oases of the Tarim Basin. Understanding these interactions is crucial to understanding the formation and spatial layout of oases, which can provide some useful guidelines for preventing desertification and protecting the fragile oasis ecosystems in the long term.
